# Allopurinol-Induced Uncommon Dermatological Emergency of Toxic Epidermal Necrolysis (TEN)

**DOI:** 10.7759/cureus.44812

**Published:** 2023-09-06

**Authors:** Umesh Kumar Pabani, Zahid Khan, Layla Ali, Shuheda K Shah, Jebriel Ali Khan

**Affiliations:** 1 Internal Medicine, Mid and South Essex NHS Foundation Trust, Southend-on-Sea, GBR; 2 Acute Medicine, Mid and South Essex NHS Foundation Trust, Southend-on-Sea, GBR; 3 Cardiology, Barts Heart Centre, London, GBR; 4 Cardiology and General Medicine, Barking, Havering and Redbridge University Hospitals NHS Trust, London, GBR; 5 Cardiology, Royal Free Hospital, London, GBR; 6 Internal Medicine, Southend University Hospital, Southend-on-Sea, GBR; 7 General Medicine, Mid and South Essex NHS Foundation Trust, Southend-on-Sea, GBR; 8 Respiratory Medicine, Great Western Hospital, Swindon, GBR

**Keywords:** allopurinol-induced ten, drug-induced ten, medication-induced ten, antibiotics cream, severe cutaneous adverse drug reactions, adverse cutaneous drug reaction, cutaneous adverse drug reaction, systemic steroids, toxic epidermal necrolysis (ten)

## Abstract

Toxic epidermal necrolysis (TEN) is a life-threatening, blistering dermatitis. It is characterized by fever and the development of mucocutaneous lesions, which lead to necrosis and sloughing of the epidermis. It is commonly triggered by medications and infections. We present the case of a 75-year-old male who presented to the hospital with a fever and widespread exfoliating skin rash involving 41% of his body surface area (BSA). He has a past medical history of gout, hypertension, asthma, and depression. He was recently started on allopurinol by his general practitioner (GP) for hyperuricemia. The condition also involved oral, eye, and pharynx mucosae. He was diagnosed with toxic epidermal necrolysis and was managed with intravenous (IV) hydrocortisone, steroid and antibiotic eye drops, and steroid and antibiotic topical creams. Due to the weak available evidence supporting the use of ciclosporin and intravenous immunoglobulins, this patient was managed with steroid use only. His rash initially worsened, but ultimately, he made a full recovery without any sequelae. The patient was reviewed in the dermatology clinic four weeks post-discharge, and he did not have any residual disease.

## Introduction

Toxic epidermal necrolysis (TEN) is a life-threatening dermatological condition characterized by blistering lesions. It is a part of the spectrum of syndromes including Stevens-Johnson syndrome (SJS) and overlap syndrome, however, with a greater percentage of total skin involvement, i.e., greater than 30% [1]. The condition also involves mucosa in different parts of the body, and in the majority of cases, two or more mucosae are affected, e.g., the oral cavity, eyes, and urogenital tract. This spectrum of dermatological diseases commonly manifests as a side effect of certain medications and infections, and a key factor in the management of the condition is the identification and cessation of the causative agent [2].

As per the British Medical Journal (BMJ) article, the United Kingdom (UK) is seeing an increasing trend in the number of adverse drug reaction (ADR)-related hospital admissions including drugs such as aspirin, warfarin, non-steroidal anti-inflammatory drugs (NSAIDs), and anti-gout medications [3]. Allopurinol is one of the most effective and commonly used methods of managing gout. However, as with any drug, it has its side effects. Although rare, allopurinol-related adverse events can be severe and are related to a high risk of morbidity. They include severe cutaneous adverse reactions (SCAR), drug reactions with eosinophilia and systemic symptoms (DRESS), TEN, SJS, and allopurinol hypersensitivity syndrome (AHS) [4]. In this case report, we present the case of a TEN reaction caused by allopurinol usage, which was successfully managed in a high-dependency unit (HDU).

## Case presentation

We present the case of a 75-year-old male who was brought to the emergency department (ED) with pyrexia and a worsening widespread skin rash over the previous 2-3 days. Onset was five days before attendance in the ED. One week ago, he was started on allopurinol by his general practitioner (GP) for gout. Otherwise, he denied any recent major illness, trauma, stress, or any change in his regular medications. He has a background of gout, hypertension, asthma, and depression. He is not known to have any allergies and only takes sertraline and losartan as regular medications.

**Figure 1:**
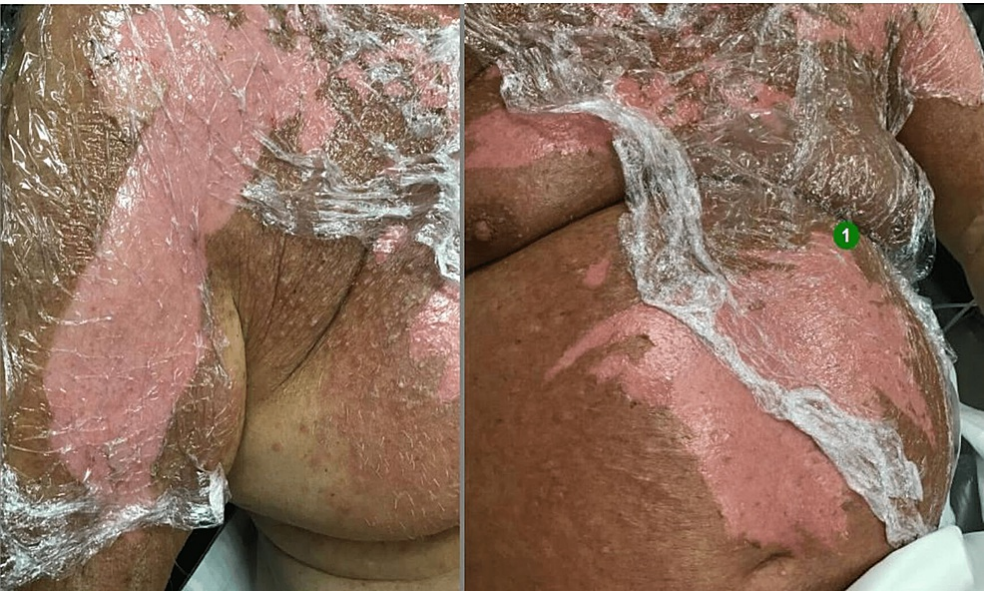
Characteristic rash of TEN in the patient TEN: toxic epidermal necrolysis

On examination, the rash involved his face, chest, abdomen, perineum, anterior thighs, shoulder, and upper arms along with mucosae of the eye, mouth, and pharynx, which approximately covered 41% of his body surface area (BSA) (calculated using Wallace rule of nines). It started as large painful blisters with associated erythema and tenderness. Blisters on his chest and abdomen had ruptured, leaving a raw bright red dermis uncovered (Figure 1). There were also a few intact fluid-filled blisters, specifically on his thighs, which later self-ruptured. The rash was associated with a fever of 39°C (recorded on admission), sore throat and odynophagia, eye pain, blurring of vision, and gritty eyes.

The patient was subsequently diagnosed with TEN based on the history of recent new drug use (allopurinol), rash starting as blisters and leaving raw dermis following rupture, rash involving more than 30% of the body surface area (BSA), rash affecting mucous membrane of at least two different sites, and co-existing inflammatory response as evident by fever. Admission blood revealed raised inflammatory markers, hyperuricemia, and acute kidney injury stage 1 (Table 1).

**Table 1:** Laboratory values of the patient during admission

Blood test	Day 1	Day 7	Normal range
Hemoglobin	111 g/L	103 g/L	133-173 g/L
White cell count	9.4 × 10^9^/L	12.6 × 10^9^/L	3.8-11 × 10^9^/L
Neutrophil count	7.86 × 10^9^/L	11.28 × 10^9^/L	1.7-7.5 × 10^9^/L
Eosinophil count	0.33 × 10^9^/L	0.01 × 10^9^/L	0.0-0.4 × 10^9^/L
Platelet count	141 × 10^9^/L	195 × 10^9^/L	150-400 × 10^9^/L
C-reactive protein	231 mg/L	23 mg/L	0-5 mg/L
Prothrombin time	11.7 seconds	13.3 seconds	10.3-13.3 seconds
Activated partial thromboplastin time	22.2 seconds	25 seconds	25.7-35.3 seconds
Amylase	371 U/L	167 U/L	28-100 U/L
Lactate dehydrogenase	352 U/L	-	240-480 U/L
Sodium	138 mmol/L	144 mmol/L	133-146 mmol/L
Potassium	4.2 mmol/L	4.4 mmol/L	3.5-5.3 mmol/L
Urea	10 mmol/L	8.1 mmol/L	2.5-7.8 mmol/L
Creatinine	139 umol/L	89 umol/L	59-104 umol/L
Albumin	34 g/L	28 g/L	35-50 g/L
Corrected calcium	2.28 mmol/L	2.20 mmol/L	2.20-2.60 mmol/L
Uric acid	573 umol/L	480 umol/L	200-430 umol/L
Alanine transaminase	47 U/L	14 U/L	<50 U/L
Glucose	4.9 mmol/L	6.4 mmol/L	<11.1 mmol/L
Bicarbonate	18 mmol/L	22.1 mmol/L	22-29 mmol/L

The patient was admitted to the high-dependency unit (HDU) due to a significant risk of complications and deterioration. His score of toxic epidermal necrolysis (Severity-of-Illness Score for Toxic Epidermal Necrolysis (SCORTEN)) on admission was 4 (given his age, blood urea nitrogen, bicarbonate level, and BSA). Since the available evidence supporting the use of intravenous (IV) immunoglobulins or ciclosporin is weak, he was managed with intravenous and topical steroids only with further supportive care to prevent complications such as dehydration and secondary infections. He was reviewed by a dermatologist and was started on intravenous hydrocortisone (100 milligrams, four times a day), topical fusidic acid, and betamethasone cream. Prophylactic antibiotics flucloxacillin 1 gram IV four times a day were given to prevent secondary infection and intravenous Hartmann’s solution was given at 125 mL/hour. Topical antiseptic dressings were also applied to cover the raw dermis. Due to eye involvement, he was seen by an ophthalmologist and was started on dexamethasone 0.1% and chloramphenicol 0.5% eye drops four times a day.

On days 3 and 4, his rash extended to his lower legs and back as part of the disease process. However, from day 7, it started to improve. He was no longer febrile with improved inflammatory markers and resolution of his acute kidney injury. The patient otherwise made an uneventful recovery and was discharged on a weaning steroids dose with a 5 mg reduction in dose every five days. He was seen in the dermatology clinic four weeks after discharge, and he made a full recovery.

## Discussion

TEN is a life-threatening, blistering dermatitis. It is characterized by fever and the development of cutaneous lesions, which lead to necrosis and sloughing of the epidermis involving more than 30% body surface area [1]. It is commonly triggered by medications and infections. SJS is a less severe condition characterized by skin detachment involving less than 10% of the body surface area [2]. In our patient, the precipitant was allopurinol, which is a xanthine oxidase inhibitor commonly prescribed for gout and hyperuricemia [3]. It has been reported as a potential cause of severe cutaneous adverse reactions (SCAR), including SJS and TEN [5]. It is essential to understand these ADRs as they can often lead to hospital admissions and can increase morbidity and mortality, as well as financial burden [4]. This case adds to existing literature, further highlighting the association between the use of allopurinol and the development of TEN.

The risk of developing SJS/TEN is highest within the initial eight weeks of drug treatment. Drugs that are used for an extended period are less likely to be associated with the onset of these conditions. Typically, the time between the start of continuous drug use and the onset of the reaction ranges from four days to four weeks [6]. The patient in this case commenced allopurinol a week before he developed the rash.

The most common drugs/drug groups associated with cases of severe cutaneous reactions include allopurinol, aromatic anti-seizure medications and lamotrigine, antibacterial sulphonamides, nevirapine, and non-steroidal anti-inflammatory drugs (NSAIDs) [7]. *Mycoplasma pneumoniae* infection is the next most common cause, particularly in children, and in one-third of TEN/SJS cases, no cause can be found [6,8]. In TEN, patients often experience a prodrome of fever and flu-like symptoms before developing skin lesions. There is usually mucosal involvement as was demonstrated in this case study. This can occur in the form of oral ulcers and ocular and urogenital inflammation and can occur before or after skin eruption [9]. Initially, the skin lesions appear as erythematous patches and then progress to blisters and sloughing. Nikolsky sign may be positive; this is demonstrated by applying some pressure to an area of the skin that is not involved in the disease process. If the area of superficial sloughing can extend, the Nikolsky sign is said to be positive [10]. The acute phase of TEN lasts 8-12 days, and re-epithelialization may begin after several days and can take up to weeks [11]. SJS/TEN can lead to serious complications including fluid loss, electrolyte imbalance, shock, renal failure, and respiratory and gastrointestinal issues [12-14]. It is essential that supportive management be directed to cover these aspects and monitor potential complications that can arise especially in the early phase of the disease.

To make the diagnosis of SJS/TEN, we rely on the clinical assessment of the patient, focusing on the history of recent drug introduction or febrile illness as discussed previously. SCORTEN can be used to predict the prognosis of SJS/TEN in an individual. This scoring system looks at seven clinical and laboratory variables (Table 2), each scoring 1.

**Table 2:** SCORTEN scale for toxic epidermal necrosis SCORTEN: Severity-of-Illness Score for Toxic Epidermal Necrolysis [15]

Risk factor	Score = 0	Score = 1
Age	<40 years	>40 years
Presence of malignancy	No	Yes
Heart rate	<120 bpm	≥120 bpm
Extent of skin detachment	<10%	≥10%
Serum bicarbonate	≥20 mmol/L	<20 mmol/L
Serum urea nitrogen	≤10 mmol/L	>10 mmol/L
Serum glucose	<13.99 mmol/L	>13.88 mmol/L

A prognostic score was used to assess the associated mortality rate of TEN. The patient being assessed in this case study scored 4 points in total, giving a mortality rate of 58.3%. One point was allocated for age being more significant than 40, hyperuricemia, 41% BSA, and low bicarbonate levels. A higher score indicates a higher risk of mortality [15].

The mainstay of management is supportive, including identifying and eliminating the causative agent, calculating the extent of skin detachment, prognostication, admitting a patient to special burns or high-dependency unit, and preventing secondary complications by implying methods such as the use of artificial membranes/dressings to cover raw dermis and early ophthalmological assessment and intervention in cases of eye involvement to prevent permanent damage. Fluid replacement is essential to prevent septic shock and hypovolemia [16]. There is limited evidence to suggest the use of systemic immunosuppressants/immunomodulating agents including systemic corticosteroids, etanercept, cyclosporin, and intravenous immune globulins (IVIG) as treatment in SJS/TEN [17]. However, more research and evidence are required to ascertain their effectiveness. The patient in our case did not receive any IVIG. He did receive intravenous hydrocortisone as well as other topical therapies. Several meta-analysis observational studies have been carried out to evaluate immunomodulatory treatments for SJS/TEN. The findings suggest that IVIG, cyclosporin, etanercept, and corticosteroids do show survival benefits. However, these studies had limitations in terms of quality [18,19]. Initially, IVIG was thought to be a potential treatment for SJS/TEN. However, studies have shown mixed results, and overall, there is insufficient evidence to indicate that IVIG reduces mortality compared to using supportive treatment only [19]. The overall mortality rate of SJS/TEN is around 25%, with TEN having a higher mortality rate. The most common causes of death include sepsis and multi-organ failure [19,20].

## Conclusions

TEN is a serious complication of certain medications, and the condition can be life-threatening if untreated. This case unquestionably highlights the importance of further research required to ascertain the best course of treatment in managing TEN, as it carries significant morbidity and mortality. TEN patients should receive treatment in the hospital, and patients should receive skin heals, supportive care including pain relief, caring for wounds, and intravenous fluids to maintain hydration. Recovery from TEN can take several months, and offending medications should be permanently stopped.
